# Case of Fatal Hepatitis Related to HEV-3 Infection in Central Italy

**DOI:** 10.3390/v16121869

**Published:** 2024-11-30

**Authors:** Monica Borghi, Alessandro Graziani, Daniele Marini, Elisabetta Madonna, Umbertina Villano, Elisabetta Suffredini, Teresa Vicenza, Elida Mataj, Roberto Bruni, Anna Rita Ciccaglione, Barbara Camilloni, Silvia Bozza

**Affiliations:** 1 Istituto Zooprofilattico Sperimentale dell’Umbria e delle Marche, 06126 Perugia, Italy; m.borghi@izsum.it; 2 Microbiology and Clinical Microbiology Section, Department of Medicine and Surgery, University of Perugia, 06132 Perugia, Italy; alessandro.graziani@dottorandi.unipg.it (A.G.); silvia.bozza@unipg.it (S.B.); 3 Microbiology Unit, Santa Maria della Misericordia Hospital, 06132 Perugia, Italy; daniele.marini@ospedale.perugia.it; 4 Department of Infectious Diseases, Unit of Viral Hepatitis and Oncovirus and Retrovirus Diseases, Istituto Superiore di Sanità, 00161 Rome, Italy; elisabetta.madonna@iss.it (E.M.); umbertina.villano@iss.it (U.V.); roberto.bruni@iss.it (R.B.); annarita.ciccaglione@iss.it (A.R.C.); 5 Department of Food Safety, Nutrition and Veterinary Public Health, Istituto Superiore di Sanità, 00161 Rome, Italy; elisabetta.suffredini@iss.it (E.S.); teresa.vicenza@iss.it (T.V.); 6 Institute of Public Health (ISHP), 1000 Tirana, Albania; elidamata@yahoo.com

**Keywords:** hepatitis E virus, genotype 3, viral hepatitis, phylogenetic analyses, foodborne disease

## Abstract

Hepatitis E virus (HEV) is a global health problem, causing an estimated 20 million infections annually. Thus, the management of HEV requires special consideration. In developed countries, hepatitis E is mainly recognized as a foodborne disease (mainly transmitted via undercooked meat consumption) that is generally caused by genotype 3 and 4 circulating in various animals, including pigs and wild boars. The current absence of officially recognized protocols for the analysis of HEV in foods and the lack of awareness of this disease among healthcare workers, together with the high percentage of asymptomatic cases, make HEV infection highly underestimated. Most HEV-3 infections in immunocompetent individuals are self-limited. Nevertheless, the possibility of serious forms of liver disease, especially in patients with co-morbidities, should be considered because it can lead to a fatal outcome. Here, we report a case of fatal hepatitis related to HEV-3 infection in a 67-year-old male patient with underlying chronic liver disease (CLD) and living in a region where a high prevalence and genetic heterogeneity of HEV-3 in wild boar has been recently demonstrated. Our case report describes the interdisciplinary approach used (from the diagnosis to the virus phylogenetic characterization) in order to improve epidemiologic HEV surveillance in central Italy.

## 1. Introduction

Viruses are considered the most common cause of foodborne disease. The World Health Organization (WHO), in 2020, estimated that hepatitis A virus (HAV) causes approximately 1.4 million infections and approximately 7000 deaths each year, while hepatitis E virus (HEV) causes 20 million infections, 3.3 million symptomatic cases, and 44,000 fatalities annually [1].

HEV, a member of the *Hepeviridae* family, is a small non-enveloped or quasi-enveloped virus with a single-stranded positive-sense ribonucleic acid (RNA) genome. The viral genome encodes for three open reading frames (ORFs), namely, ORF1, ORF2, and ORF3, but a fourth open reading frame (ORF4), embedded within ORF1 and present in genotype 1 strains only, has also been described. Among these regions, ORF1 encodes functional non-structural proteins (e.g., RNA-dependent RNA polymerase), and ORF2 encodes the highly immunogenic viral capsid protein. The antibodies against this protein have neutralizing and protective features. ORF3 encodes a functional ion channel protein that has important roles in the release of viral particles. The recently discovered ORF4 is believed to play a role in the proper functioning of HEV RNA polymerase, but the lack of this sequence in the other genotypes suggests that its real function needs to be elucidated [2,3].

HEV has mainly fecal–oral or zoonotic transmission, according to the genotypes (see below). Additionally, although rare, the parenteral transmission of HEV is also possible, for example, after whole-blood or blood products transfusion [4,5]. Phylogenetic analysis classifies HEV into eight different genotypes, of which HEV-1, HEV-2, HEV-3, and HEV-4 are responsible for disease in humans [6]. The HEV-1 and HEV-2 genotypes exclusively infect humans and are transmitted by the fecal–oral route in low-income countries, especially through contaminated water. Although these two genotypes cause self-limiting disease and are not related to cases of chronicity and/or cirrhosis, HEV-1- and HEV-2-related infections still have a substantial burden on public health in low-income countries because they occur most often in young adults and have a high incidence and severity in pregnant women, with high maternal and perinatal mortality rates [2,7,8].

HEV-3 and HEV-4 infections are mainly associated with zoonotic transmission, occurring via close contact with infected animals or through the consumption of contaminated food products (most commonly raw or undercooked meat). HEV-3 and HEV-4 infections vary widely in severity, from clinically silent to fulminant hepatitis, and they may be also responsible for extrahepatic diseases. Most HEV-3 and HEV-4 infections in immunocompetent individuals are self-limited and result only in clinically silent seroconversion. Less than 2% of those infected are symptomatic [9]. 

Nevertheless, in specific populations, such as immunosuppressed patients or individuals with underlying chronic liver disease (CLD), HEV infection is a potential trigger of acute-on-chronic liver failure [10,11].

Older individuals usually display more severe liver disease, with a higher incidence of hepatic or non-hepatic complications (15%) and acute liver failure (8–11%) [12]. In European countries, chronic hepatitis E is mostly reported in immunocompromised patients with HEV-3 infection [2,13,14]. 

In May 2022, the 75th World Health Assembly defined a new set of integrated strategies for the global health sector, to be implemented in the period 2022–2030, regarding the management of HIV, viral hepatitis, and sexually transmitted infections.

Following what was established during this assembly, many Member States, including Italy, have begun to develop national programs and strategies aimed at achieving the intended objective, through community training interventions and prevention, diagnosis, and treatment actions.

In Italy, HEV infection is subject to notification through the infectious disease information system (PREMAL), based on reports from doctors who alert the Public Health and Hygiene Service every time a diagnosis of acute hepatitis E is made [15]. 

Since 2007, it has been possible to notify cases of acute viral hepatitis also through an integrated epidemiological surveillance known as Sistema Epidemiologico Integrato dell’Epatite Virale Acuta (SEIEVA).

SEIEVA describes and monitors the trends of the various forms of acute viral hepatitis, differentiated by specific type, throughout Italy. The integrated analysis of the information collected with the epidemiological questionnaires allows for the estimation of the incidence of disease and the relative contribution of the different risk factors. This also allows for the definition of preventive measures to be prioritized and for the monitoring of the effects of the various prevention programs.

Thanks to this surveillance system, between February 2021 and August 2023, 16 cases of acute hepatitis E were diagnosed and monitored in Umbria (Central Italy), and here, we describe a case of fatal hepatitis related to HEV-3 infection in a 67-year-old male patient with underlying CLD.

## 2. Case Description

### 2.1. Clinical Presentation

A 67-year-old male, presenting with asthenia and loss of weight, accompanied by the presence of jaundice, hyperchromic urine, and hypocolic feces was admitted to the Internal Medicine Section of the Perugia Hospital (Umbria, Italy) on 3 February 2022. He denied abdominal pain, vomiting, diarrhoea, fever, and other associated symptoms. His medical history included a previous diagnosis of type II diabetes mellitus treated at home with oral hypoglycemic therapy, hypertensive cardiopathy with mitro-aortic valvulopathy, hypercholesterolemia associated with obesity (Body Mass Index = 31), mild congenital interventricular septal defect, and a history of knee prosthesis and inguinal hernioplasty.

At admission, SARS-CoV-2 and hemato-biochemical tests were performed. SARS-CoV-2 molecular and antigenic tests were negative upon admission and during the stay in the Internal Medicine Section. The results of the hemato-biochemical tests are reported in Table 1.

Based on the laboratory results, gastroenterological and nephrological consultation were requested.Abdominal ultrasound revealed hepatosplenomegaly with hypertrophy of the right and caudate lobes of the liver. In addition, the presence of collateral circles (probably esophageal varices) and ectasia of the left gastric vein were identified. Pulsed Wave Doppler (PW) examination highlighted the presence of hepatoportal flow with significantly reduced average velocity. The gallbladder, normally distended, appeared filled with biliary sludge. Ascitic effusion of moderate size was detected, estimated to be at least 2000 cc. 

A diagnosis of liver cirrhosis with portal hypertension was made, with a probable dysmetabolic genesis, aggravated by a picture of acute renal failure (ARF) with tubular damage due to hyperbilirubinemia. From the anamnestic collection, the exotoxin and iatrogenic hypothesis of the liver pathology was excluded. 

Major causes of hepatitis, including HAV, HBV, and HCV, *Cytomegalovirus*, and Epstein–Barr virus, were excluded. Meanwhile, from the serological analysis for major hepatotropic viruses, on 4 February 2022, positivity for HEV emerged. The IgM and IgG, detected using VIDAS^®^ Anti-HEV IgM and VIDAS^®^ Anti-HEV IgG kits (bioMérieux, France), were 39.4 S/Co and >10.00 U/mL, respectively (IgM cut-off = 1.0 S/Co, IgG cut-off = 0.56 U/mL). 

A detailed medical history excluded recent trips abroad or contact with travellers in the 9 weeks preceding the onset of hepatitis. The patient also denied a history of transfusion while reporting frequent consumption of wild boar meat, as he was a hunter.

As required by the protocol, the anti-HEV IgM positive serum of the patient was sent to the Laboratory for Viral Hepatitis, Oncovirus, and Retrovirus Diseases (Istituto Superiore di Sanità, ISS), where further analyses were conducted to confirm the diagnosis of acute hepatitis E. The serological analyses confirmed the presence of anti-HEV IgG and IgM (Wantai HEV-IgG and Wantai HEV-IgM ELISA assays, Beijing WANTAI Biological Pharmacy Enterprise Co. Ltd., Beijing, China). In parallel, molecular investigation detected the presence of the viral RNA (10.000 IU/mL) using a Real-Time PCR assay (RealStar HEV RT-PCR Kit 2.0, Altona Diagnostics GmbH, Hamburg, Germany), according to the manufacturer instructions. 

Considering the diagnosis of HEV infection, gastroenterological consultation was requested. The ultrasound examination of the abdomen highlighted an unknown full-blown liver cirrhosis with portal hypertension. 

Based on the overall clinical picture of the patient, the liver failure attributable to acute HEV infection was not interpreted as acute liver failure (ALF) but as acute-on-chronic failure (ACLF) with dysmetabolic genesis, complicated by portal hypertension. His Mayo End-stage Liver Disease (MELD) score, a prognostic scoring system based on laboratory parameters, used to predict 3-month mortality due to liver disease, was 38. The MELD score ranges from 6 to 40, and the higher the score, the higher the 3-month mortality related to liver disease. Therefore, for our patient, the prognosis was poor. 

**Figure 1:**
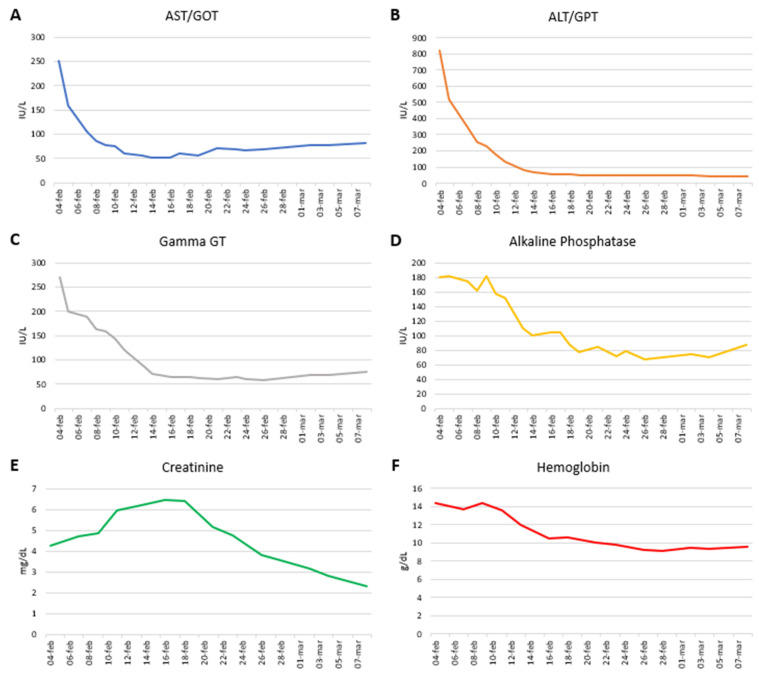
Graphic presentation of patient’s liver enzyme levels throughout the hospitalization. Trend of liver enzymes from the day after the hospital admission (4 February 2022) to the day before the discharge (7 March 2022). (**A**) Aspartate aminotransferase/glutamic oxaloacetic transaminase (AST/GOT); (**B**) alanine aminotransferase/glutamic–pyruvic transaminase (ALT/GPT); (**C**) gamma glutamyltransferase (Gamma GT); (**D**) alkaline phosphatase; (**E**) creatinine; (**F**) hemoglobin.

During the hospital stay, a new basal-bolus insulin therapy was started, obtaining a good glycemic profile. Additionally, an antibiotic prophylaxis with Meropenem was started, and vitamin K and albumin were administered to compensate for liver dysfunction. The patient remained paucisymptomatic throughout his hospitalization. Gradually, there was a partial recovery of liver function (Figure 1). 

Following repeated episodes of vomiting after meals, the patient was readmitted to the emergency room and monitored for one day on 15 March 2022. Patient was apyretic and had no other symptoms. Biological examinations notably revealed a modest neutrophilic leucocytosis (WBC = 9.87 × 10^3^/uL, 84.1% of neutrophils, INR = 2.43, azotemia = 90 mg/dL, creatinine = 1.71 mg/dL, CKD-EPI eGFR = 40.8 mL/min/1.73 mq, total bilirubin = 42.63 mg/dL, direct bilirubin = 17.41 mg/dL, AST/GOT = 117 IU/L, ALT/GPT = 61 IU/L, CRP = 2 mg/dL). A SARS-CoV-2 test was negative. HEV serology was repeated, showing IgM and IgG values of 37.9 S/Co and >10.00 U/mL, respectively. After 24 h of observation, blood tests showed a spontaneous improvement in leucocytosis. Considering the clinical picture with a treatable and non-painful abdomen, apyrexia, and the positive course of the hospital stay, the patient was transferred to Ancona Transplant Centre and evaluated for the antiviral therapy and liver transplant. HEV RNA was tested in serum and stools during the stay; it was not detected in either sample. Antiviral therapy was not initiated and transplant was denied because of the patient’s age and the presence of significant comorbidities. 

Subsequently, on 28 May 2022, following a picture of liver cirrhosis in the phase of ascitic decompensation, asthenia, and new episodes of vomiting, a new hospitalization was necessary. During the hospitalization, a new serological investigation was performed, and anti-HEV IgM and anti-HEV IgG levels were still high (IgM = 14.2 S/Co and IgG > 10.00 U/mL). Urine culture resulted positive for *Escherichia coli* (>100.000 CFU/mL), and antibiotic therapy with Fosfomycin (3 gr/die per 3 days) was prescribed. Despite the treatments provided, due to the slow and irreversible deterioration of the clinical picture and following the complications that arose in relation to the HEV infection, the patient’s death was declared on 19 June 2022.

### 2.2. HEV Genotype/Subtype and Phylogenetic Analyses

To characterize the virus, the extracted RNA was subjected to nested PCR amplification of an ORF2 fragment (nucleotide position 5948-6513 in the reference sequence, accession number M73218) of the viral genome, according to a previously described procedure [16]. The nested PCR product (size: 566 nt) was then subjected to double-strand sequencing by a Sanger Sequencing kit (Applied Biosystems by Thermo Fisher Scientific) and an automated sequencer (SeqStudio Genetic Analyzer, Applied Biosystems by Thermo Fisher Scientific). After removal of the primer sequence ends from the raw output sequences, the final consensus sequence (size: 493 nt) was subjected to phylogenetic analysis with subtype 3 reference sequences as well as with HEV-3 sequences obtained from wild boars collected in Umbria in 2021–2022 [17].

**Figure 2:**
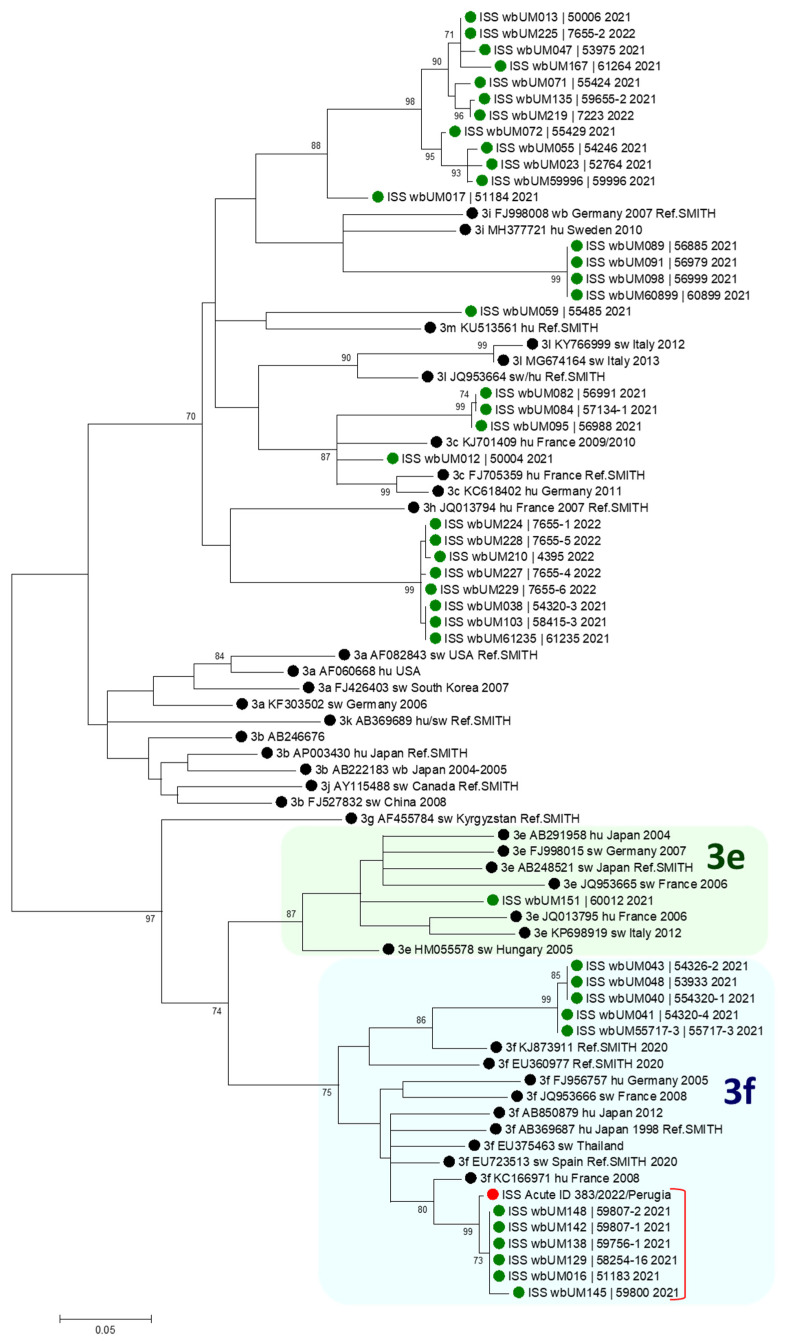
Phylogenetic analysis. Phylogenetic tree resulting from analysis of a 361 nt region shared by the HEV sequence from the case, sequences from wild boars sampled in 2021–2022 in Umbria, and reference sequences representing subtypes 3a to 3m [18]. A maximum likelihood approach was applied, with a TN93+G+I evolutionary model preliminarily estimated by the Model tool in MEGA. The red circle highlights the sequence from the patient, the black circles mark reference sequences, and the green circles mark sequences from wild boars from the region of Umbria. The suffix “Ref. SMITH” at the end of some reference sequence names marks those reference sequences recommended by an international group of experts as the best representative subtype references [18]. The 3e and 3f clades are highlighted by green and light blue shading; a red square bracket delimits the cluster that includes the patient sequence and six wild boar sequences.

Figure 2 shows the resulting phylogenetic tree. The patient sequence (red circle) is placed in the 3f clade, well separated from the 3e clade by a statistically supported node (bootstrap > 70); so, it can be assigned to subtype 3f. 

It is noteworthy that the sequence also forms a separate cluster with a group of six HEV sequences from wild boars sampled in November–December 2021 in places within 60 kilometers from Perugia (Città di Castello 52 Km; Ascagnano, 22 Km; Ficareto, nearby Todi, 50 Km; San Giustino, 61 Km). Significantly, the patient had his residence in Perugia, he was a wild boar hunter, and he reported frequent consumption of wild boar meat. Five wild boar sequences of this cluster are identical to each other; the patient sequence shows three nt differences vs. these five wild boar sequences and seven nt differences vs. the sixth remaining sequence.

Although the patient and wild boar sequences are not identical, sequence comparison shows that the patient HEV strain was genetically highly related to viruses circulating in wild boars in the same geographical area and in the same period in which the patient hunted wild boars.

### 2.3. Food Analyses

Concurrently with the investigation conducted on the patient, experts from the Prevention, Veterinary Health, and Food Safety Service of the Umbria Region, in collaboration with the Experimental Zooprophylactic Institute of Umbria and Marche “Togo-Rosati” (IZSUM), and together with the National Reference Laboratory for Foodborne Viruses, recovered from the patient’s household and tested food matrices suspected to be vehicles of the infection. Eight packages of frozen wild boar meat and four packages of home-made frozen sausages, made with mixed wild boar/pig minced meat, were recovered from the patient’s freezer. Analyses were performed following an in-house standardized protocol, including lysis of sample (1 g), according to Szabo et al. [19], nucleic acid extraction using a bioMérieux NucliSens MiniMag system, and HEV detection by RT-qPCR, as described in Di Pasquale et al. [20]. HEV nucleic acid was detected at a low concentration (~10 genome copies/g) in two sausage packages, confirming the patient’s hunting activity as a plausible source of exposure. Subsequent attempts to amplify a viral genome fragment by nested PCR for sequencing and comparison with the HEV sequence from the patient were unsuccessful, probably due to the low viral load in the samples.

## 3. Discussion

Many studies, mostly outside Europe, report HEV infection as one of the most important causes of acute-on-chronic liver failure (ACLF) in patients with CLD, which is associated with high mortality rates [21,22]. Indeed, HEV infection is potentially fatal in patients with underlying liver disease [23].

Data collected through SEIEVA confirmed what is already known from the literature [24]: the category most at risk of contracting the infection is that made up of males aged >50 years. For instance, in our region, of the 16 positive subjects with HEV infection between February 2021 and August 2023, 13 (81.25%) were male and had an average age of 54 years (range 39–83), including the patient reported in this case report.

Our patient had a regular lifestyle before the infection, despite having cirrhosis of metabolic origin. Thus, HEV infection had a significant negative impact on his quality of life and on the management of the numerous preexisting pathological conditions. Moreover, as recently reported by Abravanel et al., cardiovascular comorbidities represent an important risk factor for severe HEV infection and are associated with higher hospitalization [25]. 

Considering the rapidly changing epidemiological situation and based on the European Food Safety Authority (EFSA) statement that the number of HEV infection cases in Europe shows a steadily increasing trend [4], an active surveillance plan was activated in the Umbria region. HEV has thus become a special watchdog.

Following reports from hospital clinicians, thanks to the SEIEVA, it was possible to quickly activate a series of epidemiological investigations that allowed the Unit of Viral Hepatitis and Oncovirus and Retrovirus diseases (at ISS) to promptly isolate viral RNA from an early sample of patient serum. This should be emphasized because, as is well known, the diagnosis of acute hepatitis E in humans is based on the specific detection, in the patient’s serum, of IgM antibodies directed against HEV. In case of IgM positivity of the serologic test, the laboratory also proceeds with molecular tests for direct detection of the virus in the patient’s blood.

Of note is the fact that the detection of viral RNA in serum may be difficult because very often the diagnosis of HEV infection is made at a late stage and the viremia is under the detectable level [26].

This case of infection must be considered autochthonous because the patient had reported no travel in HEV hyperendemic areas before the onset of hepatitis. Moreover, he referred to habitual consumption of wild boar meat, which is considered an important risk factor especially in our region, where Beikpour et al. [17] recently demonstrated a high prevalence and genetic heterogeneity of genotype 3 HEV in wild boar. The laboratory results of the present study (sequence comparison, phylogenetic analysis, detection of HEV in home-made sausages in the patient freezer), together with previous data collected in the same region [27], overall suggest, although do not definitively prove, HEV transmission associated with the hunting activity of the patient, either through slaughtering wild boars and handling their meat or through consumption of food originating from them.

Based on the analysis carried out and the real risk of an increase in cases, hepatitis E should be considered as an emerging public health issue in our region too. 

Underestimating HEV infection in European and American patients is the most common mistake that clinicians make, due to the predominantly asymptomatic course of the disease. Nevertheless, the possibility of sudden deterioration of liver function, especially in patients with CLD, should be considered regardless of their travel history or their conscious exposure to known potential risk factors. The European Association for the Study of the Liver (EASL) suggests that patients with unexplained CLD flares should be tested for hepatitis E [28].

As reported by the ISS, in the last few years, the number of cases of hepatitis E has slightly exceeded cases of acute hepatitis C. In 2023, hepatitis E was the third most frequent cause of viral hepatitis in Italy [29].

To date, neither vaccines nor specific antiviral treatments against HEV are available. The control of HEV at a territorial level can be guaranteed exclusively through the implementation of constant prevention and monitoring programs, which must involve all those operating in the sector of public health, veterinary health, food safety and environmental protection with a One-Health approach. 

In developed countries, HEV is principally recognized as a foodborne disease, and while an ISO standard method for the detection of HEV in meat products is under development, the current absence of officially recognized protocols for the analysis of HEV in foods hinder the implementation of targeted control plans in the EU.

Furthermore, the lack of awareness of this disease among healthcare workers and the high percentage of asymptomatic cases make HEV infection highly underestimated.

HEV can cause very serious forms of liver disease and be associated with the onset of severe forms of chronic hepatitis in immunosuppressed subjects or with previous diagnoses of liver cirrhosis. The present case report documents that the occurrence of HEV infection in a patient with CLD and significant comorbidities may lead to an exacerbation of the clinical conditions, leading to a fatal outcome. Taking into account that antiviral drugs, such as ribavirin, in cases of anemia and/or renal failure should be dosed with caution [28], prevention of infection remains the only protection tool.

## Figures and Tables

**Table 1:** Laboratory results at admission.

Laboratory Measurement	Result	Unit of Measurement	Normal Range
White Blood Cells (WBCs)	8.03	×10^3^/uL	(3.60–9.60)
Red Blood Cells (RBCs)	4.60	×10^6^/uL	(4.30–5.80)
Hemoglobin	14.1	g/dL	(13.0–17.0)
Hematocrit	40.6	%	(38.0–52.0)
MCV	88.3	fL	(82.0–97.0)
MCH	30.7	pg	(27.0–33.0)
MCHC	34.7	g/dL	(32.0–36.0)
RDW	* 14.9	%	(11.6–14.5)
Platelets	289	×1000/UL	(140–440)
MPV	10.1	fL	(8.0–13.0)
Lymphocytes	* 15.7	%	(20.5–51.5)
Monocytes	* 12.0	%	(1.0–10.0)
Neutrophils	69.1	%	(42.0–75.0)
Eosinophils	2.2	%	(0.0–5.0)
Basophils	1.0	%	(0.0–1.0)
I.N.R	* 1.41		(0.80–1.20)
Glycemia	* 146	mg/dL	(74–106)
Azotemia	* 138	mg/dL	(17–43)
Creatinine	* 3.03	mg/dL	(0.62–1.18)
CKD-EPI eGFR	* 20.4	mL/min/1.73 mq	(>60.0)
Total Bilirubin	* 33.71	mg/dL	(0.30–1.20)
Direct Bilirubin	* 15.55	mg/dL	(0.00–0.20)
AST/GOT	* 459	IU/L	(0–50)
ALT/GPT	* 1244	IU/L	(0–50)
Gamma GT	* 290	IU/L	(0–55)
Alkaline Phosphatase	* 202	IU/L	(30–120)
Sodium	* 135	mEq/L	(136–146)
Potassium	4.0	mEq/L	(3.5–5.1)
Amylase	45	IU/L	(28–100)

* Values out of normal range; Note: Highly jaundiced specimen.

## Data Availability

The data are available upon request from the corresponding author. The data are not publicly available because of privacy or ethical restrictions.
